# Conserved and unique transcriptional features of pharyngeal arches in the skate (*Leucoraja erinacea*) and evolution of the jaw

**DOI:** 10.1093/molbev/msab123

**Published:** 2021-04-27

**Authors:** Christine Hirschberger, Victoria A Sleight, Katharine E Criswell, Stephen J Clark, J Andrew Gillis

**Affiliations:** 1 Department of Zoology, University of Cambridge, Cambridge, United Kingdom; 2 School of Biological Sciences, University of Aberdeen, Aberdeen, United Kingdom; 3 Babraham Institute, Cambridge, United Kingdom; 4 Marine Biological Laboratory, Woods Hole, MA, USA

**Keywords:** evo-devo, jaw, development, pharyngeal arch, patterning, serial homology

## Abstract

The origin of the jaw is a long-standing problem in vertebrate evolutionary biology. Classical hypotheses of serial homology propose that the upper and lower jaw evolved through modifications of dorsal and ventral gill arch skeletal elements, respectively. If the jaw and gill arches are derived members of a primitive branchial series, we predict that they would share common developmental patterning mechanisms. Using candidate and RNAseq/differential gene expression analyses, we find broad conservation of dorsoventral (DV) patterning mechanisms within the developing mandibular, hyoid, and gill arches of a cartilaginous fish, the skate (*Leucoraja erinacea*). Shared features include expression of genes encoding members of the ventralizing BMP and endothelin signaling pathways and their effectors, the joint markers nkx3.2 and gdf5 and prochondrogenic transcription factor barx1, and the dorsal territory marker pou3f3. Additionally, we find that mesenchymal expression of *eya1*/*six1* is an ancestral feature of the mandibular arch of jawed vertebrates, whereas differences in notch signaling distinguish the mandibular and gill arches in skate. Comparative transcriptomic analyses of mandibular and gill arch tissues reveal additional genes differentially expressed along the DV axis of the pharyngeal arches, including *scamp5* as a novel marker of the dorsal mandibular arch, as well as distinct transcriptional features of mandibular and gill arch muscle progenitors and developing gill buds. Taken together, our findings reveal conserved patterning mechanisms in the pharyngeal arches of jawed vertebrates, consistent with serial homology of their skeletal derivatives, as well as unique transcriptional features that may underpin distinct jaw and gill arch morphologies.

## Introduction

**Fig. 1.**
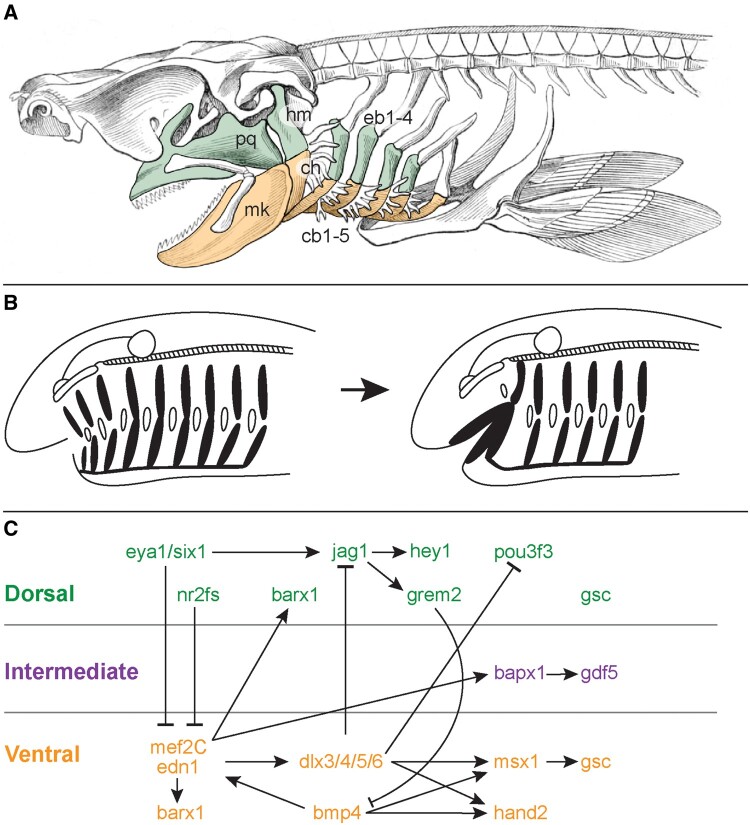
Anatomy, evolution, and patterning of the pharyngeal endoskeleton. (*A*) Shark head skeleton illustrating an hypothesis of serial homology of the jaw and gill arch skeleton. Upper (dorsal) jaw, hyoid, and gill arches are green, lower (ventral) jaw, hyoid, and gill arches are orange (schematic modified from Owen 1866). (*B*) Representative textbook scenario of jaw origin by transformation of an anterior gill arch (redrawn from Janvier 1996 and references therein). (*C*) Signaling pathways and downstream effectors patterning the DV axis of the jaw, as established largely from studies in mouse and zebrafish (redrawn largely after Cerny et al. [2010] and Medeiros and Crump [2012]). *cb1-5*, ceratobranchials 1-5; *ch*, ceratohyal; *eb1-5*, epibranchials 1-5; *hm*, hyomandibula; *mk*, Meckel’s cartilage; *pq*, palatoquadrate.

The jaw is an iconic example of anatomical innovation, and a uniting feature of the jawed vertebrate (gnathostome) crown group (Gans and Northcutt 1983; Mallatt 1996; Northcutt 2005). Over a century ago, the anatomist Karl Gegenbaur proposed a scenario of serial homology, whereby the upper and lower jaw arose through modifications of the dorsal and ventral elements of an anterior gill arch (Gegenbaur 1878—fig. 1*A*). This hypothesis was based largely on the strikingly similar anatomical organization of the jaw and gill arches of cartilaginous fishes (sharks, skates, and rays), and has since gained wide acceptance as a textbook scenario of jaw origin (Goodrich 1930; de Beer 1971; Romer 1966; Carroll 1988; though see Janvier 1996 and Miyashita 2016 for review and critical discussion of this hypothesis—fig. 1*B*).

The endoskeletal elements of the jaw and gills develop from pharyngeal arches—transient, segmentally repeated columns of mesoderm and neural-crest-derived mesenchyme encased by epithelium in the embryonic vertebrate head (Graham 2003). These embryonic tissues give rise to different elements of the craniofacial anatomy: head musculature forms from the pharyngeal arch core mesoderm, skeletal and connective tissue elements derived from neural crest and mesodermal mesenchyme, epidermal covering and sensory neurons derived from the ectodermal epithelium, and the inner lining of the pharynx and associated endocrine organs derived from the endoderm. In gnathostome “fishes,” the first (mandibular) pharyngeal arch gives rise to the jaw skeleton, the second (hyoid) arch gives rise to a gill bearing arch that also functions, in some lineages, to suspend the jaw from the braincase, and a variable number of gill arches give rise to the skeletal support of the gills. The skeletal derivatives of the pharyngeal arches of gnathostomes were ancestrally segmented, principally, dorsoventrally into the palatoquadrate and Meckel’s cartilage in the jaw, the hyomandibula and ceratohyal in the hyoid arch, and the epi- and ceratobranchial elements in the gill arches (de Beer 1971; Janvier, 1996—fig. 1*A*).

Cyclostomes (lampreys and hagfishes) are the most proximate extant sister group of gnathostomes (Heimberg et al. 2010), and they possess a mandibular arch-derived velar skeleton that is neither supportive of a gill, nor organized into segments or subcomponents with clear serial homologues in the more caudal, gill-supporting arches. Thus, although the last common ancestor of the vertebrate crown group may well have possessed a mandibular arch-derived skeleton that was, morphologically, differentiated from that of the more caudal arches, whether the jaw of gnathostomes evolved from a more cyclostome-like condition (subsequently converging on a gill-arch like endoskeletal organization—i.e., primitive anisomery, *sensu*Miyashita and Diogo 2016), or from a general dorsoventrally segmented skeletal condition that was ancestrally shared by the mandibular and gill arches (i.e., primitive polyisomery) remains unresolved. Currently, a series of transitional fossils showing the stepwise acquisition of the jaw along the gnathostome stem is lacking, and this gap in the fossil record has made it difficult to support or refute hypotheses of jaw-gill arch serial homology with paleontological data. But elements of such hypotheses are, nevertheless, testable from a developmental perspective. Over the past several decades, concepts of serial homology have evolved to center largely around the iterative deployment or sharing of conserved developmental mechanisms (e.g., Van Valen 1982; Roth 1984; Wagner 1989, 2007, 2014). If the parallel anatomical organization of the gnathostome jaw and gill arch skeleton is a product of serial homology, we predict that these elements would be delineated by shared patterning mechanisms—and, conversely, that their anatomical differences may be attributable to arch-specific variations on a core, conserved developmental program.

Studies in zebrafish and mouse have revealed a network of signaling interactions and transcription factors that are key to the development and patterning of the dorsal and ventral segments of the jaw in bony vertebrates (fig. 1*C*). Briefly, Endothelin-1 (edn1) and bone morphogenetic protein 4 (bmp4) signaling from ventral mandibular arch epithelium and mesoderm promotes ventral expression of *dlx5/6*, *hand2*, and *msx1/2* and imparts lower jaw identity (Clouthier et al. 1998; Beverdam et al. 2002; Depew et al. 2002; Miller et al. 2003; Yanagisawa et al. 2003; Ozeki et al. 2004; Alexander et al. 2011; Zuniga et al. 2011). Conversely, notch signaling (Zuniga et al. 2010; Barske et al. 2016) and *six1* expression (Tavares et al. 2017) promote dorsal arch identity, with Six1 repressing transcription of *edn1*. Dorsal mandibular and hyoid arch territories are broadly marked by expression of *pou3f3* (Jeong et al. 2008; Askary et al. 2017). Within the dorsal territory of the mandibular arch, the upper (maxillary) component of the jaw is specified by nr2f nuclear receptors, which promote osteogenic fate within neural-crest-derived mesenchyme, and which are, themselves, negatively transcriptionally regulated by endothelin signaling (the latter promoting chondrogenic fate within mesenchyme of the ventral mandibular arch—Barske et al. 2018). Finally, the jaw joint is specified at the interface of these upper and lower jaw gene expression domains, with the presumptive joint marked by the expression of *bapx1/nkx3.2* (Miller et al. 2003; Lukas and Olsson 2018) and *gdf5* (Miller et al. 2003), and flanked by expression of the prochondrogenic (and joint-repressing) transcription factor *barx1* (Nichols et al. 2013).

Taken together, these signaling interactions and transcription factors establish a dorsoventral (DV) code of combinatorial gene expression that confers axial identity on the mandibular and hyoid arch skeleton of bony fishes (fig. 1*C*), though whether/which of these mechanisms were primitively shared between the mandibular, hyoid, and gill arches of gnathostomes remains unclear. We, and others, have previously shown that nested expression of the *dlx* family of transcription factors, a key regulator of DV axial identity in the mandibular arch (Beverdam et al. 2002; Depew et al. 2002, 2005; Talbot et al. 2010), was primitively shared across all pharyngeal arches in gnathostomes (Compagnucci et al. 2013; Debiais-Thibaud et al. 2013; Gillis et al. 2013), and that dorsal and ventral domains of *dlx* gene expression delineate the principal segments of the jaw and gill arch skeleton in a conserved manner in a chondrichthyan, the skate (*Leucoraja erinacea—*Gillis et al. 2013). These findings are consistent with hypotheses of serial homology of the palatoquadrate/Meckel’s cartilage and epi-/ceratobranchial gill arch elements, respectively, although the degree of conservation or divergence of upstream signals and downstream effectors of this “*dlx* code” in the mandibular and gill arches has not been fully investigated.

To test the hypothesis that the jaw and gill arches are patterned by a shared transcriptional network, we have investigated the molecular development of the pharyngeal arches in the skate. This group has retained the primitive dorsoventrally segmented organization of the gnathostome pharyngeal endoskeleton (i.e., a jaw and gill arch skeleton that is segmented into prominent palatoquadrate/Meckel’s cartilage epi-/ceratobranchial elements, respectively—Mallatt 1996; Gillis et al. 2012), and, through comparison with its sister group, the bony fishes, allows us to infer anatomical and developmental conditions in the last common ancestor of gnathostomes. Using a combination of candidate gene and comparative transcriptomic approaches, we find that the transcriptional network patterning the DV axis of the developing jaw in bony fishes is largely conserved and shared by the mandibular, hyoid, and gill arches of skate, consistent with the hypothesis of jaw-gill arch serial homology. We further resolve dorsal mesenchymal expression of *six1* and *eya1* as a primitive and unique feature of the mandibular arch, we report *scamp5* as a novel marker of the dorsal territory of the mandibular arch, and we report transcriptional differences associated with progenitors of jaw and gill arch-specific musculature and gill primordia. Taken together, our findings point to a conserved gene regulatory network underlying the primitively shared organization of the gnathostome mandibular, hyoid, and gill arch skeleton, and highlight additional transcriptional features that correlate with the developmental and anatomical diversification of jaws and gill arches within gnathostomes.

## Results and Discussion

### Conservation of Ventral Gene Expression Patterns in the Skate Mandibular, Hyoid, and Gill Arches

In mouse (Kurihara et al. 1994; Clouthier et al. 1998; Ozeki et al. 2004) and in zebrafish (Miller et al. 2000; Kimmel et al. 2007), *edn1* is expressed in ventral and intermediate mandibular and hyoid arch epithelium, and this edn1 signal is transduced within the adjacent arch mesenchyme through its receptor, ednra, and its downstream effector mef2C (Miller et al. 2007; Nair et al. 2007; Sato et al. 2008). *bmp4* is similarly expressed in ventral arch epithelium in mouse (Liu et al. 2005) and in zebrafish (Alexander et al. 2011), where its ventral patterning function is restricted by intermediate expression of *grem2*, which encodes a secreted Bmp antagonist (Zuniga et al. 2011). Together, edn1 and bmp4 signaling promote ventral mesenchymal expression of *hand2*, *msx1*, and ventral *dlx* genes, and confer lower jaw identity (Thomas et al. 1998; Yanagisawa et al. 2003; Zuniga et al. 2011; Funato et al. 2016).

**Fig. 2.**
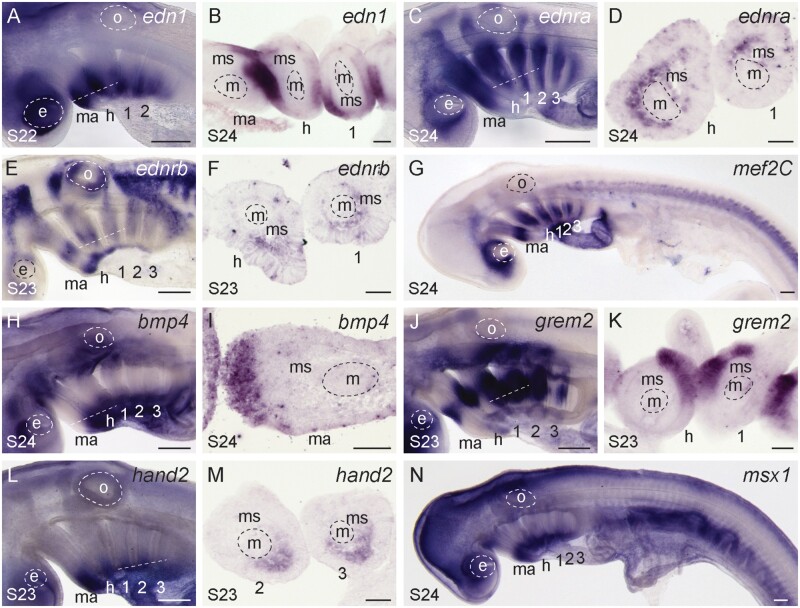
Conservation of ventral gene expression patterns in the skate mandibular, hyoid, and gill arches. (*A*) At S22, *edn1* is expressed in the ventral domain of all pharyngeal arches, with transcripts localizing to the (*B*) pharyngeal epithelium. (*C*) *ednra* is expressed along the entire DV axis of the pharyngeal arches, within (*D*) the mesenchyme. (*E*) *ednrb* is expressed in migrating neural crest streams, and also in distinct intermediate and ventral domains within (*F*) pharyngeal arch mesenchyme. (*G*) *mef2C* is expressed in the ventral and intermediate domains of all pharyngeal arches. (*H*) *bmp4* is expressed in ventral pharyngeal arch (*I*) epithelium, and (*J*) *grem2* is expressed in intermediate pharyngeal arch (*K*) epithelium. (*L*) *hand2* is expressed in the ventral (*M*) mesenchyme of each pharyngeal arch. (*N*) *msx1* is expressed ventrally in all pharyngeal arches. All sections are horizontal, with approximate plane indicated by a white dashed line in the corresponding wholemount. *1*, *2*, *3*, gill arches 1–3; *e*, eye; *h*, hyoid arch; *m*, mesoderm; *ma*, mandibular arch; *ms*, mesenchyme; *o*, otic vesicle. Scale bars: 400 µm in wholemounts, 25 µm in section images.

We carried out a series of mRNA in situ hybridization (ISH) experiments to test for shared expression of ventral patterning factors in the pharyngeal arches of skate embryos. We found that *edn1* is expressed in the ventral/intermediate epithelium of the mandibular, hyoid, and gill arches (fig. 2*A* and *B*), whereas *ednra* is expressed throughout the mesenchyme of all pharyngeal arches (fig. 2*C* and *D*). Notably, analysis of *edn1* expression in an extended developmental series of skate embryos (supplementary fig. S1, Supplementary Material online) revealed no expression in the core mesoderm of the pharyngeal arches, with the exception of very low-level and spatially restricted expression within the ventral-intermediate gill arch mesoderm at S26/S27 (supplementary fig. S1*F*, Supplementary Material online). This differs considerably from the strong pharyngeal arch core mesodermal expression of *edn1* in mouse (Maemura et al. 1996), chick (Nataf et al. 1998), zebrafish (Miller et al. 2000), and the jawless lamprey (Square et al. 2016), and points to a likely loss or substantial reduction of mesodermal *end1* expression in cartilaginous fishes. We additionally tested for expression of the gene encoding another endothelin receptor, *ednrb*. Although expression of *ednrb* genes have not been reported in pharyngeal arch mesenchyme of other gnathostome model systems (reviewed by Pla and Larue 2003), skate embryos exhibit shared expression of *ednrb* in ventral and intermediate mesenchyme across all pharyngeal arches (fig. 2*E* and *F*), hinting at conservation within gnathostomes of a skeletal patterning function of *ednrb* that has so far only been described in the lamprey (*Petromyzon marinus—*Square et al. 2020). We also found shared expression of *mef2C* in the ventral/intermediate domain of all pharyngeal arches (fig. 2*G*). It has been demonstrated that *mef2C* is a transcriptional target of edn1 signaling in cranial neural-crest-derived mesenchyme (Miller et al. 2007), and so our findings point to shared *edn1* signaling between epithelium and mesenchyme of all pharyngeal arches in skate.

We also tested for expression of bmp signaling components in skate pharyngeal arches, and found shared *bmp4* expression in the ventral epithelium of all arches (fig. 2*H* and *I*). Dorsal to this *bmp4* domain, we observe shared intermediate/dorsal expression of *grem2* in the mandibular, hyoid, and gill arch epithelium (fig. 2*J* and *K*). This *grem2* expression is similar, in terms of position along the DV axis, to what has been previously reported in zebrafish. However, *grem2* expression differs between skate and zebrafish in terms of tissue localization, with epithelial expression in the former and mesenchymal expression in the latter (Zuniga et al. 2011). Finally, we detect shared expression of *hand2* and *msx1* in the ventral mesenchyme of all pharyngeal arches (fig. 2*L*–*N*). Taken together, our findings point to conservation of ventral pharyngeal arch patterning mechanisms between bony and cartilaginous fishes, and across the mandibular, hyoid, and gill arches of the skate.

### Conserved and Divergent Dorsal Expression of Dorsal Patterning Genes in the Skate Mandibular, Hyoid, and Gill Arches

In mouse, eya1 and six1 function in craniofacial development (Xu et al. 1999; Laclef et al. 2003; Ozaki et al. 2004) and are coexpressed in the upper jaw primordium of the mandibular arch, where they inhibit expression of *edn1* and induce expression of the notch signaling component *jag1* (Tavares et al. 2017). In zebrafish, *jag1b* and *hey1* are expressed in the dorsal mesenchyme of the mandibular and hyoid arches and in pouch endoderm, whereas *notch2* is expressed more widely throughout the pharyngeal arches (Zuniga et al. 2010). Notch signaling through *jag1b* and *hey1* promotes dorsal arch identity and restricts the expression of intermediate and ventral patterning genes, including *dlx3b/5a/6a*, *msxe*, *nkx3.2*, and *barx1* (Zuniga et al. 2010; Barske et al. 2016). In zebrafish, *dlx2a* is also expressed throughout the DV mesenchyme axis of pharyngeal arches, and together with *dlx1a* functions to specify dorsal identity (Talbot et al. 2010) and, in mouse, to positively regulate the dorsal expression of another upper jaw marker within the arch mesenchyme, *pou3f3* (Jeong et al. 2008).

**Fig. 3.**
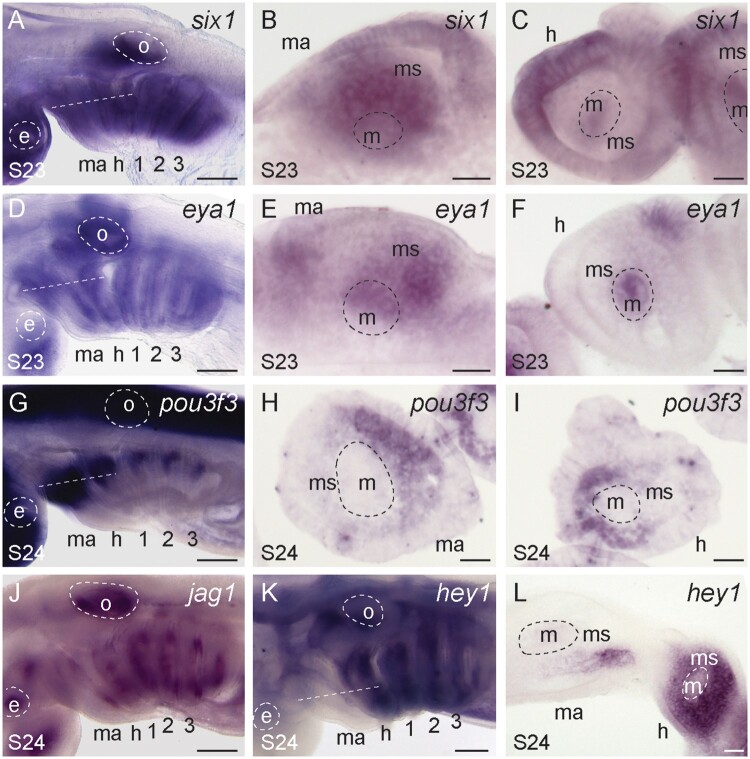
Conserved and divergent dorsal gene expression patterns in the skate mandibular, hyoid, and gill arches. (*A*) *six1* is expressed in the (*B*) mesenchyme, core mesoderm and epithelium of the mandibular arch, and in the (*C*) core mesoderm and epithelium of the hyoid and gill arches. Similarly, (*D*) *eya1* is expressed in the (*E*) mesenchyme, core mesoderm and epithelium of the mandibular arch, and in the (*F*) core mesoderm and epithelium of the hyoid and gill arches. (*G*) *pou3f3* is expressed in the dorsal mesenchyme of the (*H*) mandibular, (*I*) hyoid and gill arches. (*J*) *jag1* is expressed in the mandibular, hyoid, and gill arches, though (*K*) the notch signaling readout *hey1* is expressed (*L*) in a very restricted pattern within the mandibular arch mesenchyme, but broadly throughout the hyoid and gill arch mesenchyme. All sections are horizontal, with approximate plane indicated by a white dashed line in the corresponding wholemount. *1*, *2*, *3*, gill arches 1–3; *e*, eye; *h*, hyoid arch; *m*, mesoderm; *ma*, mandibular arch; *ms*, mesenchyme; *o*, otic vesicle. Scale bars: 400 µm in wholemounts, 25 µm in section images.

To test for conservation of dorsal patterning factors in the pharyngeal arches of the skate, we first characterized the expression of the transcription factors *eya1*, *six1*, and *pou3f3* by ISH. We found that *six1* (fig. 3*A*–*C*) and *eya1* (fig. 3*D*–*F*) are both expressed broadly in the mandibular, hyoid, and gill arches in skate. However, although *six1* and *eya1* expression in the epithelium and mesodermal core is shared across the mandibular (fig. 3*B* and *E*), hyoid, and gill arches (fig. 3*C* and *F*), mesenchymal expression of these factors is uniquely observed in the dorsal mandibular arch (fig. 3*B* and *E*). Our findings are consistent with *six1* expression reported in mouse (Tavares et al. 2017) and chick (Fonseca et al. 2017), and point to an ancestral role for *eya1/six1* in patterning the upper jaw skeleton of gnathostomes. In contrast, *pou3f3* is expressed in the dorsal mesenchyme of the mandibular, hyoid, and gill arches (fig. 3*G*–*I*), indicating a likely shared role in dorsal patterning across all pharyngeal arches.

We next tested for expression of genes encoding the notch signaling components jag1 and hey1. We observe *jag1* expression in the hyoid and gill arches of skate, but not in the mandibular arch (with the exception of very restricted expression in the posterior mandibular arch epithelium—fig. 3*J*). In line with this, we also detect strong expression of *hey1* (a notch signaling readout) throughout the mesenchyme of the hyoid and gill arches, but only very restricted expression within a subdomain of the posterior mandibular arch mesenchyme (fig. 3*K* and *L* and supplementary fig. S2*A*–*F*, Supplementary Material online). These observations differ from patterns previously reported in zebrafish, both in terms of DV extent of expression (i.e., expression along the entire DV extent of the arch in skate, as opposed to the dorsal localization seen in zebrafish), and the near exclusion of mesenchymal *hey1* expression from the mandibular arch in skate. It is possible that the dorsal arch patterning function of jag1 signaling is an ancestral feature of the gnathostome mandibular arch that has been lost or reduced in skate, or that this mechanism is a derived feature of bony fishes. Gene expression data for notch signaling components in the pharyngeal arches of cyclostomes (lampreys and hagfishes) are needed to resolve this.

### Conservation of Joint Gene Expression Patterns in the Skate Mandibular, Hyoid, and Gill Arches

**Fig. 4.**
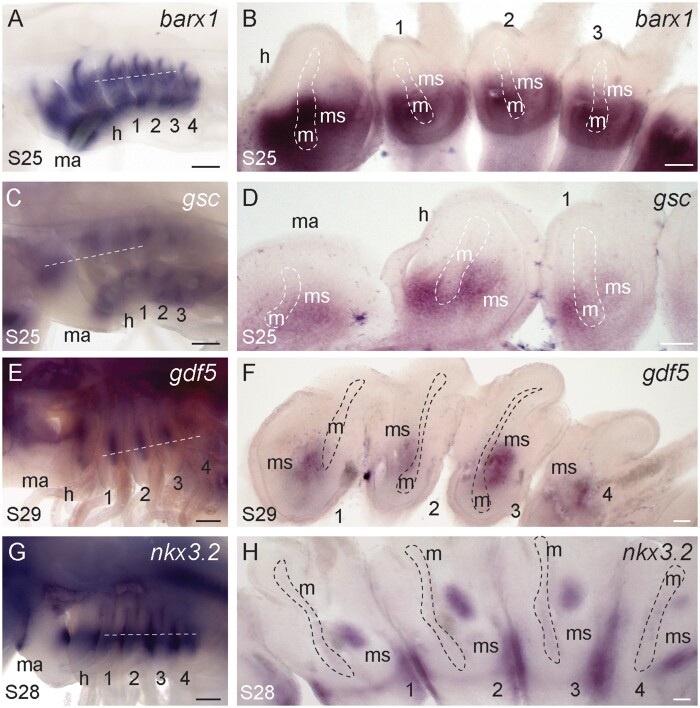
Conserved expression of joint markers and prochondrogenic transcription factors in the skate mandibular, hyoid, and gill arches. (*A*) *barx1* is expressed in dorsal and ventral (*B*) mesenchyme across all pharyngeal arches, in a pattern that flanks the presumptive joint domain. (*C*) *gsc* is also expressed in dorsal and ventral (*D*) mesenchyme domains of all pharyngeal arches, excluding the intermediate, presumptive joint domains. (*E*) *gdf5* is subsequently expressed in the intermediate (*F*) mesenchyme of all pharyngeal arches. (*G*) *nkx3.2* is expressed in the intermediate (*H*) mesenchyme and epithelium of all pharyngeal arches. All sections are horizontal, with approximate plane indicated by a white dashed line in the corresponding wholemount. *1*, *2*, *3*, gill arches 1–3; *e*, eye; *h*, hyoid arch; *m*, mesoderm; *ma*, mandibular arch; *ms*, mesenchyme; *o*, otic vesicle. Scale bars: 400 µm in wholemounts, 25 µm in section images.

In bony fishes, the jaw joint is specified by expression of genes encoding the transcription factor nkx3.2 and the secreted signaling molecule gdf5, and is flanked by expression of genes encoding the prochondrogenic transcription factor barx1, as well as gsc (Newman et al. 1997; Trumpp et al. 1999; Miller et al. 2003; Tucker et al. 2004; Wilson and Tucker 2004; Nichols et al. 2013; Lukas and Olsson 2018). In skate, we observe apparently shared mesenchymal expression of *barx1* (fig. 4*A* and *B*) and *gsc* (fig. 4*C* and *D*) in the dorsal and ventral domains of the mandibular, hyoid, and gill arches, and later, complementary mesenchymal expression of *gdf5* (fig. 4*E* and *F*) and *nkx3.2* (fig. 4*G* and *H*) in the intermediate region of all arches. These expression patterns are consistent with conservation of the prochondrogenic function of *barx1*, the joint-flanking expression of *gsc*, and the joint patterning function of *nkx3.2* and *gdf5*, in cartilaginous fishes.

A previous study of axial patterning gene expression in the pharyngeal arches of the jawless lamprey reported broad conservation of *dlx*, *hand*, and *msx* expression across all pharyngeal arches, but a conspicuous absence of *bapx* and *gdf* expression in the intermediate region of the first arch. These observations led to the suggestion that co-option of these joint patterning factors to the intermediate region of the mandibular arch, on top of a pre-existing and deeply conserved DV patterning program, was key to the evolutionary origin of the jaw (Cerny et al. 2010). Our findings are consistent with acquisition of intermediate *nkx3.2* and *gdf5* expression as a key step in the origin of the jaw joint, but suggest that this developmental mechanism was not primitively mandibular arch specific, but rather a conserved mechanism specifying joint fate in the skeleton of the mandibular, hyoid, and gill arches of gnathostomes.

### Comparative Transcriptomics Reveals Additional Mandibular and Gill Arch DV Patterning Genes

**Fig. 5.**
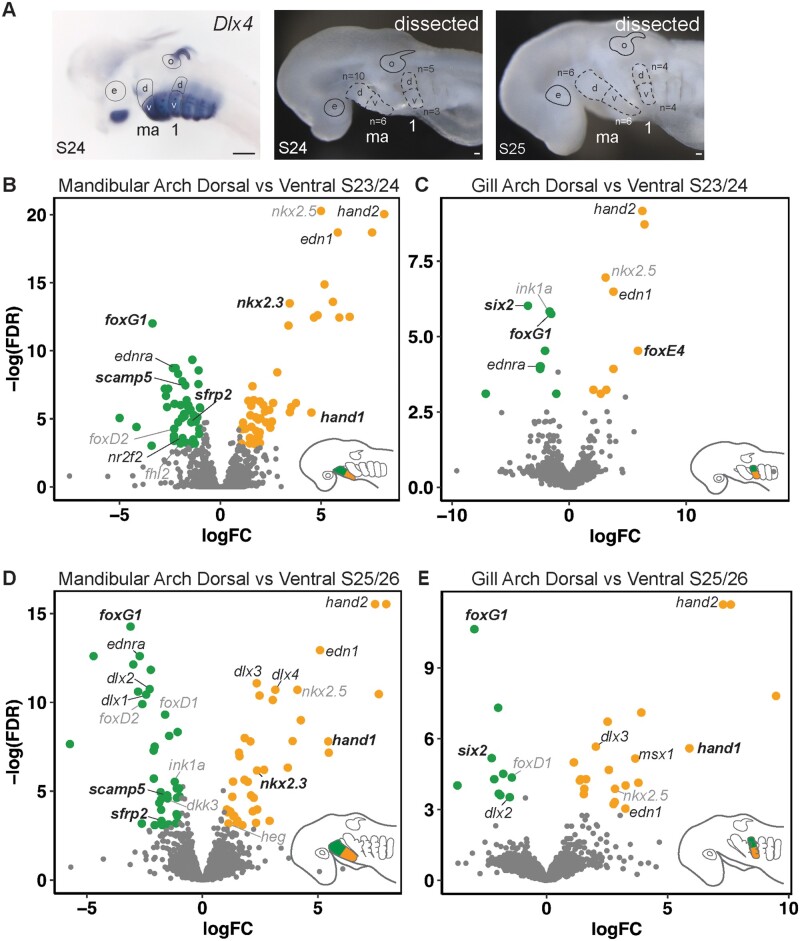
De novo transcriptome and differential gene expression analysis of dorsal and ventral domains of skate pharyngeal arches. (*A*) Demarcation of dorsal and ventral domains of the mandibular and gill arch based on previously published *Dlx* gene expression (Gillis et al. 2013). Dorsal and ventral domains of the mandibular arch and gill arch 1 were collected by manual dissection from skate embryos at S23/S24 and S25/S26. Volcano plots illustrate genes that are significantly differentially expressed within the dorsal and ventral domains of the (*B*) mandibular arch at S23/S24, (*C*) gill arch 1 at S23/S24, (*D*) the mandibular arch at S25/S26 and (*E*) gill arch 1 at S25/S26. Genes with established roles in pharyngeal arch axial patterning are in simple italics, additional genes for which we provide in situ validation are in bold italics, and additional factors highlighted by our analysis but not validated by mRNA in situ hybridization are in gray italics. *1*, gill arch 1; *d*, dorsal; *e*, eye; *ma*, mandibular arch; *o*, otic vesicle, *v*, ventral. Scale bars: black 400 µm, white 25 µm.

In an attempt to discover additional factors involved in DV patterning of the pharyngeal skeleton, we performed a comparative transcriptomic and differential gene expression analysis of upper and lower jaw and gill arch progenitors from skate embryos from S23 to S26. It is during these stages that DV axial identity is established within skate pharyngeal arches, as evidenced by nested expression within pharyngeal arches of the *dlx* family of transcription factors (Gillis et al. 2013), and by expression of the known axial patterning candidate genes characterized above. We manually dissected dorsal and ventral domains of the mandibular arch and gill arch 1 of S23/S24 and S25/S26 skate embryos (based on morphological landmarks correlating with dorsal and ventral *Dlx* code expression, after Gillis et al. 2013—fig. 5*A*), and performed RNA extraction, library preparation, and RNAseq for each half-arch. After de novo transcriptome assembly, we conducted within-arch comparisons of gene expression levels between dorsal and ventral domains of the mandibular arch and gill arch 1, and across-arch comparisons of gene expression levels between dorsal mandibular and dorsal gill arch domains, and between ventral mandibular and ventral gill arch gill arch domains (fig. 5*B*–*E* and supplementary fig. S3*B*–*E*, Supplementary Material online).

We identified a number of transcripts as differentially expressed, defined as greater than a 2-fold change between tissue types with an adjusted *P* value less than 0.05 (log2-fold changes [log2FC] > 1, *P* value adjusted using Benjamin–Hochberd method < 0.05), within and between arch types at S23–S24 and S25–S26 (supplementary table S2, Supplementary Material online). Our ability to identify differentially expressed transcripts within and between arches using this approach was corroborated by the correct identification of known or expected genes within the appropriate spatial territory—for example, *hand2*, *edn1*, and *dlx3/4* were identified as differentially expressed within ventral territories (fig. 5*B* and *D*), *nr2f2* was identified as enriched in the dorsal mandibular arch (fig. 5*B*) and *otx2* and *hox* genes were identified as differentially expressed within the mandibular and gill arch territories, respectively (supplementary fig. S3*B*–*E*, Supplementary Material online). To further biologically validate some of the findings of our analysis, we selected up to eight of the topmost differentially expressed transcription factors or signaling pathway components per comparison (excluding those already queried by our candidate gene approach or those with well-known functions in axial patterning of the pharyngeal skeleton), and attempted to clone fragments for in situ gene expressions analysis (supplementary table S3, Supplementary Material online—complete lists of differentially expressed transcripts from each comparison are provided in supplementary tables S4–S11, Supplementary Material online). Out of 37 uniquely identified genes, we generated riboprobes for an additional 15 candidates, and we tested spatial expression of these candidates by mRNA ISH.

**Fig. 6.**
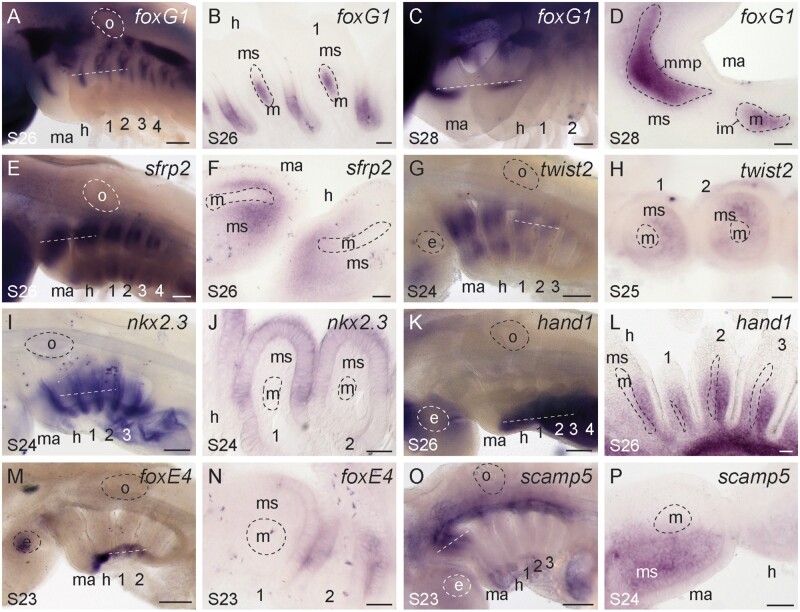
Additional genes exhibiting polarized expression along the DV axis of skate pharyngeal arches. (*A*) *foxG1* is expressed in dorsal (*B*) pharyngeal arch epithelium and core mesoderm of skate mandibular, hyoid, and gill arches at S26, and (*C*) in discrete dorsal and ventral (*D*) mesoderm-derived muscle progenitors within the mandibular arch at S28. (*E*) *sfrp2* is expressed in dorsal and ventral (*F*) mesenchyme of each pharyngeal arch. Similarly, (*G*) *twist2* is expressed in dorsal and ventral (*H*) mesenchyme of each pharyngeal arch. (*I*) *nkx2.3* is expressed in the ventral and intermediate (*J*) epithelium of each pharyngeal arch. (*K*) *hand1* transcripts localize to the ventral (*L*) mesenchyme of each pharyngeal arch. (*M*) *foxE4* is expressed in the ventral extreme of the pharyngeal region, (*N*) with transcripts localizing to the epithelium. (*O*) *scamp5* is expressed in the dorsal (*P*) mesenchyme of the mandibular arch, as well as in the ventral-most territory of all pharyngeal arches. All sections are horizontal, with approximate plane indicated by a white dashed line in the corresponding wholemount, with the exception of (*D*), which is an oblique section through the mandibular arch. *1*, *2*, *3*, *4*, gill arches 1–4; *e*, eye; *h*, hyoid arch; *ma*, mandibular arch; *m*, mesoderm; *ms*, mesenchyme; *o*, otic vesicle. Scale bars: 400 µm in wholemounts, 25 µm in section images.

We observed *foxG1* expression in the dorsal domains of the mandibular, hyoid, and gill arches in skate. In mouse, *foxG1* functions in the morphogenesis of the forebrain (Tao and Lai 1992; Dou et al. 1999; Hanashima et al. 2002), but it is also expressed in the epithelium and mesodermal core of the pharyngeal arches (Hébert and McConnell 2000; Tavares et al. 2017), and has recently been shown to play a role in neurocranial and pharyngeal skeletal development (Compagnucci and Depew 2020). In skate, we find that *foxG1* is initially expressed strongly in the dorsal epithelium and dorsal mesodermal core of each pharyngeal arch at S26 (fig. 6*A* and *B*). Subsequently, *foxG1* is strongly expressed in an additional ventral domain in the core of the mandibular arch, and at lower levels within distinct ventral domains of the hyoid and gill arch mesodermal cores, at S27/S28 (fig. 6*C* and *D* and supplementary fig. S4, Supplementary Material online). The discrete dorsal and ventral domains of *foxG1* expression within the skate mandibular arch appear to correspond with the masticatory muscle plate (which will further subdivide into the constrictor dorsalis and the adductor mandibulae) and the intermandibularis, respectively (Edgeworth 1935; supplementary fig. S4, Supplementary Material online), though cranial muscle homologies of cartilaginous fishes (and batoids, in particular) are complex, and not fully resolved (Miyake et al. 1992). Conversely, the dorsal and ventral domains of *foxG1* expression within the core of the hyoid and gill arches (supplementary fig. S4*C*–*E*, Supplementary Material online) are established while this tissue still exists as a continuous mesodermally derived “muscle plate” (supplementary fig. S4*C*′–*E*′, Supplementary Material online). Edgeworth’s (1935) seminal work on vertebrate cranial muscle development documents the iterative subdivision of an initially continuous mesodermal muscle plate within the core of each pharyngeal arch into distinct dorsal and ventral domains, with subsequent division into discrete muscles. Within zebrafish, the homeodomain transcription factor engrailed marks the mesodermal progenitors and differentiated myocytes of the dorsal mandibular arch-derived levator arcus palatini and dilator opercula (Hatta et al. 1990), whereas *edn1* is expressed in a ventral subdivision of the core mesoderm of the mandibular and hyoid arches (Miller et al. 2000). These gene expression patterns may determine mesodermal segment identity within the framework of Edgeworth’s model of pharyngeal arch muscle development (Miyake et al. 1992; Kimmel et al. 2001). Although we did not observe ventral mesodermal *edn1* expression in the pharyngeal arches of the skate (see above, and supplementary fig. S1, Supplementary Material online), our expression data from skate point to *foxG1* as an additional molecular correlate of Edgeworth’s model of muscle plate subdivision, potentially delineating the dorsal and ventral muscle plate subdivisions of each pharyngeal arch prior to and immediately following their separation.

We additionally found that *sfrp2* (fig. 6*E*) and *twist2* (fig. 6*G*) are expressed in a discontiguous pattern, in the dorsal and ventral domains of skate pharyngeal arches. In chick, *sfrp2* is expressed in migrating cranial neural crest cells (Terry et al. 2000), whereas in mouse, it is expressed in the mesenchyme of the maxillary and mandibular domains of the mandibular arch (Leimeister et al. 1998). *sfrp2* is also expressed in the pharyngeal arches in zebrafish (Tendeng and Houart 2006), where RNAseq experiments found it to be enriched in cranial neural crest cells of the dorsal mandibular and hyoid arches (Askary et al. 2017). However, wholemount fluorescent ISH in zebrafish detected *sfrp2* expression only in the dorsal mesoderm, and TALEN and CRISPR induced early frameshift mutations in this gene did not lead to any observable skeletal craniofacial phenotypes (Askary et al. 2017). *twist2* is a basic helix-loop-helix transcription factor that is expressed in the dermis, cranial mesenchyme, pharyngeal arches, and tongue of the mouse (Li et al. 1995), and in the mesenchyme of the mandibular and hyoid arches in chick (Scaal et al. 2001). Human nonsense mutations in *twist2* are linked to Setleis syndrome, a focal facial dermal dysplasia, and *twist2* knockout mice exhibit a similar facial phenotype (Tukel et al. 2010). In skate, we observed mesenchymal expression of both *sfrp2* (fig. 6*E* and *F*) and *twist2* (fig. 6*G* and *H*) in the dorsal and ventral mesenchyme of all pharyngeal arches, in patterns reminiscent of the prochondrogenic gene *barx1*, suggesting a possible role for these genes in the regulation of chondrogenesis.

Among genes with predicted expression in ventral pharyngeal arch territories, we found shared ventral expression of *nkx2.3*, *foxE4*, and *hand1* across all pharyngeal arches in skate. *nkx2.3* is expressed in the endodermal lining of the pharynx in frog, mouse, and zebrafish (Evans et al. 1995; Lee et al. 1996; Biben et al. 2004), and in skate, we find conservation of this pharyngeal endodermal expression (though with ventral endodermal localization of *nkx2.3* transcripts at S24—fig. 6*I* and *J*). In mouse, *hand1* functions in cardiac morphogenesis (Srivastava et al. 1995; Riley et al. 1998), but is also expressed in the ventral mesenchyme of the pharyngeal arches (Clouthier et al. 2000). Targeted deletion of *hand1* alone does not result in craniofacial defects, though ablation of *hand1* on a *hand2* heterozygous background results in ventral midline defects within the jaw skeleton, suggesting a dosage-dependent role for *hand* genes in mandibular skeletal patterning (Barbosa et al. 2007). Skate *hand1* is expressed in the ventral mesenchyme of each pharyngeal arch (fig. 6*K* and *L*), in a pattern largely overlapping with the ventral mesenchymal expression of *hand2*, consistent with an ancestral combinatorial role for *Hand* genes patterning the ventral pharyngeal arch skeleton of gnathostomes. Finally, *foxE4* is expressed in the pharyngeal endoderm of nonteleost ray-finned fishes (Minarik et al. 2017), and in the endostyle (an endodermally derived secretory organ and putative evolutionary antecendent of the thyroid gland) in nonvertebrate chordates (Yu et al. 2002; Hiruta et al. 2005). In skate, *foxE4* expression is conserved in ventral pharyngeal endoderm (fig. 6*M* and *N*), pointing to an ancestral role for this transcription factor in pharyngeal endodermal patterning, and possible also in thyroid development.

Our analyses highlighted several genes that were differentially expressed between pharyngeal arch territories, but that were not immediately annotated by BLAST against UniProt/Swiss-Prot, and that required further manual annotation by BLASTing against the larger NCBI nonredundant (nr) database. Among these was *scamp5*, which encodes a secretory carrier membrane protein expressed in the synaptic vesicles of neuroendocrine tissues (Fernández-Chacón and Südhof 2000; Han et al. 2009), and falls within the same topologically associated domain as single nucleotide polymorphisms associated with orofacial clefting in humans (Carlson et al. 2019). In skate, *scamp5* is expressed in dorsal mandibular arch mesenchyme, with a lower level of expression also detectable in the ventral-most territory of all pharyngeal arches (fig. 6*O* and *P* and supplementary fig. S2*G* and *H*, Supplementary Material online). Although *scamp5* has never been previously implicated in pharyngeal arch skeletal patterning, the above observations, combined with our novel in situ expression in skate, highlight this gene as a promising candidate for further study. Expression analyses and functional characterization in bony fish model systems will reveal whether the expression patterns we report here are general features of gnathostomes, or derived features of cartilaginous fishes, and possible undiscovered roles for *scamp5* in craniofacial skeletal development.

### Distinct Gene Expression Features within Mandibular and Gill Arch Mesodermal Muscle Progenitors

The mesodermal cores of vertebrate pharyngeal arches derive from both cranial paraxial and lateral splanchnic mesodermal subpopulations, and give rise to the branchiomeric musculature—that is, the muscles of mastication and facial expression in mammals, and the muscles of the jaw and gill arches in fishes (Tzahor and Evans 2011; Ziermann and Diogo 2019; Sleight and Gillis 2020). Although expression of some elements of the pharyngeal myogenic developmental program, such as *Tbx1* (Kelly et al. 2004), *Islet-1* (Nathan et al. 2008), *Lhx2* (Harel et al. 2012), myosin heavy chain (Ziermann et al. 2017), and *MyoD* (Schilling and Kimmel 1997; Poopalasundaram et al. 2019) are shared across the mesodermal cores of multiple pharyngeal arches, other gene expression features are differentially required for the specification of distinct arch-derived muscular features. For example, it has been shown in mouse that *Pitx2* expression within the core mesoderm of the mandibular arch is required for specification of jaw musculature—in part through positive regulation of core mesodermal *Six2* expression—but not for specification of hyoid arch musculature (Shih et al. 2007). It therefore appears as though pharyngeal arch myogenesis is regulated by a core transcriptional program, with additional arch-specific gene expression directing specific branchiomeric muscle identities.

**Fig. 7.**
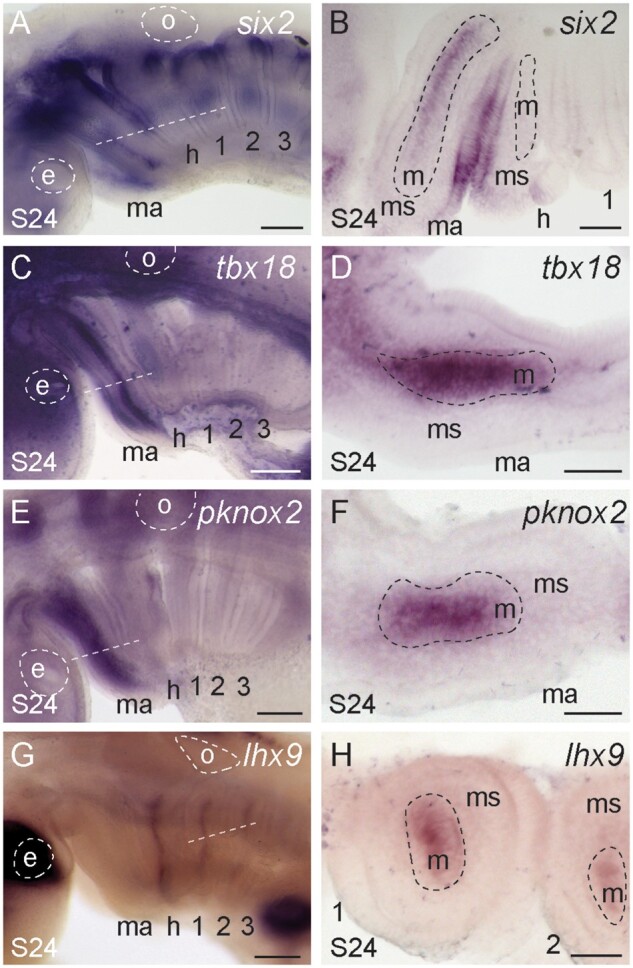
Distinct gene expression features of mandibular and hyoid/gill arch muscle progenitors. (*A*, *B*) *six2*, (*C*, *D*) *tbx18*, and (*E*, *F*) *pknox2* are expressed in the core mesoderm of the mandibular arch. *six2* is also expressed in the dorsal epithelium of each pharyngeal arch. (*G*, *H*) *lhx9* is expressed in the core mesoderm of the hyoid and gill arches. All sections are horizontal, with approximate plane indicated by a white dashed line in the corresponding wholemount. *1*, *2*, *3*, *4*, gill arches 1–4; *e*, eye; *h*, hyoid arch; *ma*, mandibular arch; *m*, mesoderm; *ms*, mesenchyme; *o*, otic vesicle. Scale bars: 400 µm in wholemounts, 25 µm in section images.

Our differential expression analyses identified *six2* as enriched in the skate mandibular arch, and in situ validation confirmed its expression in the mesodermal core of the mandibular arch at S24 (as well as in the dorsal epithelium of each pharyngeal arch—fig. 7*A* and *B*). We have also identified *tbx18* (fig. 7*C* and *D*) and *pknox*2 (fig. 7*E* and *F*) as markers of the mesodermal core of the mandibular arch at S24. *Tbx18* expression within the mandibular arch has previously been reported in mouse (Kraus et al. 2001), zebrafish (Begemann et al. 2002), and chick (Haenig and Kispert 2004), whereas *Pknox2* expression has previously been reported from microarray analysis of the mouse mandibular arch (Feng et al. 2009). However, neither *Tbx18* nor *Pknox2* has yet been implicated in the development of mandibular arch-derived musculature. Interestingly, our analyses also revealed *lhx9* as a marker of the mesodermal core of the hyoid and gill arches, but not the mandibular arch (fig. 7*G* and *H*)—a feature so far unreported in any other taxon. Taken together, these findings highlight an ancestral role for *six2* in patterning mandibular arch-derived musculature in jawed vertebrates, possibly in conjunction/parallel with *tbx18* and *pknox2*, as well as *lhx9* as a novel marker of hyoid and gill arch muscle progenitors.

### Gene Expression Features of Presumptive Gill Epithelium and External Gill Buds

**Fig. 8.**
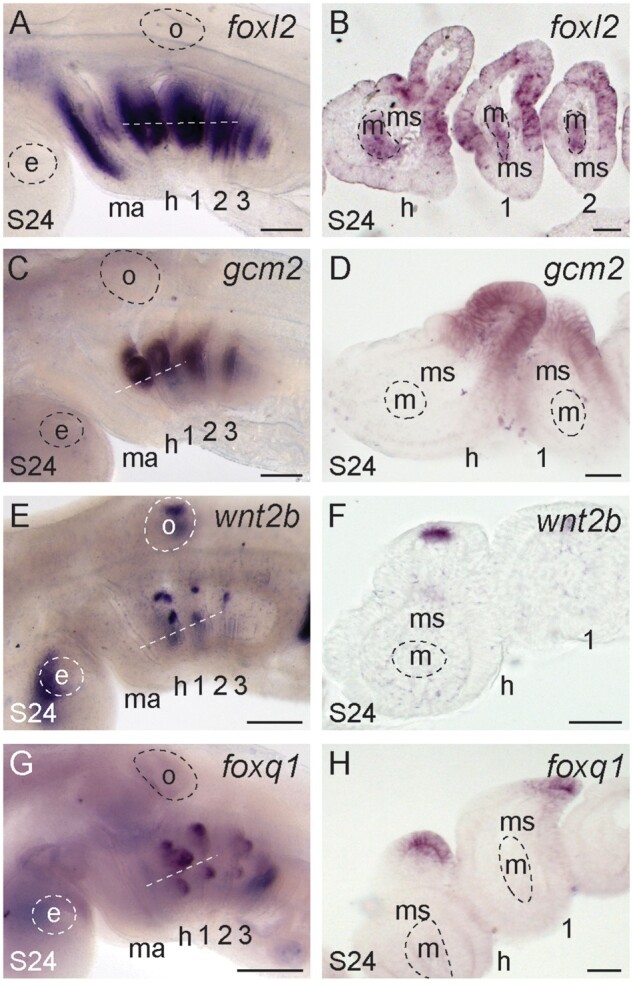
Conserved and novel molecular markers of gill development. (*A*, B) *foxl2* is expressed in the gill-forming epithelium and core mesoderm of all pharyngeal arches in skate at S24. (*C*, *D*) *gcm2* is expressed throughout the developing gill buds of the hyoid and gill arches, whereas (*E*, *F*) *wnt2b* and (*G*, *H*) *foxq1* are expressed in the tips of developing gill buds. All sections are horizontal, with approximate plane indicated by a white dashed line in the corresponding wholemount. *1*, *2*, *3*, *4*, gill arches 1–4; *e*, eye; *h*, hyoid arch; *ma*, mandibular arch; *m*, mesoderm; *ms*, mesenchyme; *o*, otic vesicle. Scale bars: 400 µm in wholemounts, 25 µm in section images.

The gills of fishes derive from the endodermal epithelium of the hyoid and gill arches (Warga and Nüsslein-Volhard 1999; Gillis and Tidswell 2017; Hockman et al. 2017). In skate, gills form initially as a series of transient embryonic external gill filaments, which are eventually remodeled and resorbed into internal gill lamellae (Pelster and Bemis 1992). Our differential expression analysis revealed a number of genes to be differentially expressed between the mandibular arch and gill arch 1, some of which proved, through in situ validation, to be markers of developing gills. In skate, we observed expression of *foxl2* in the gill-forming endodermal epithelium and developing gill buds of all pharyngeal arches (including the presumptive spiracular pseudobranch primordium—i.e., the precursors of the vestigial gill lamellae of the mandibular arch), as well as in the core mesoderm of each pharyngeal arch (fig. 8*A* and *B*). These expression patterns are consistent with previous reports of *foxL2* expression from mouse (Jeong et al. 2008; Marongiu et al. 2015) and the shark, *Scyliorhinus canicula* (Wotton et al. 2007). We additionally observe expression of *gcm2* throughout the developing gill buds of the hyoid and gill arches (fig. 8*C* and *D*), as well as expression of *wnt2b* (fig. 8*E* and *F*) and *foxQ1* (fig. 8*G* and *H*) in the tips of the developing gill buds. *gcm2* is expressed in the developing gills of shark and zebrafish (Hogan et al. 2004; Okabe and Graham 2004), and is therefore a conserved marker of developing gills in gnathostomes. However, there are no previous reports of *wnt2b* or *foxq1* expression during gill development in other taxa, pointing to a possible novel role for these factors in driving outgrowth of external gill filaments.

### Mandibular and Gill Arch Serial Homology and Evolution of the Jaw

**Fig. 9.**
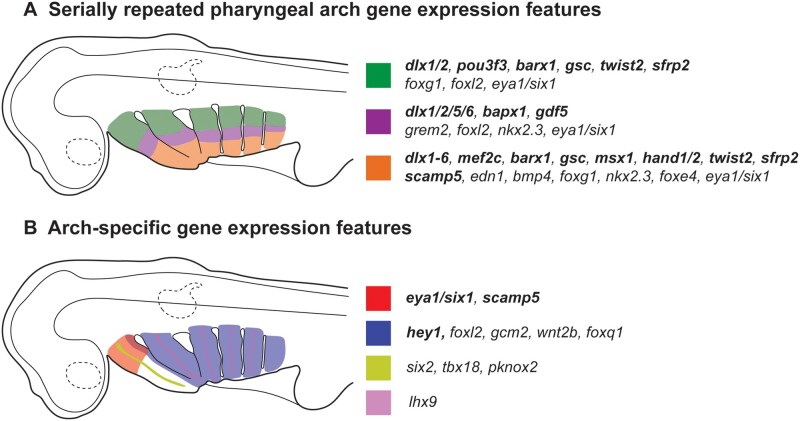
Summary of polarized gene expression patterns within skate pharyngeal arches. (*A*) Gene expression patterns that are serially repeated across the mandibular, hyoid, and gill arches in skate. We propose that these features comprise an ancestral core pharyngeal arch DV patterning program for gnathostomes, and underlie serial homology of the jaw, hyoid, and gill arch skeleton. For schematic purposes, serially repeated gene expression patterns are classified as belonging to one of three broad territories (dorsal, intermediate, or ventral). (*B*) Gene expression features that are unique to one or more pharyngeal arches in skate. Bold italics indicates genes that are expressed in pharyngeal arch mesenchyme, whereas regular italics indicates genes that are expressed in pharyngeal arch mesoderm and/or epithelium. For details of expression patterns and tissue specificity, please see text.

Our combination of candidate and differential gene expression analysis has revealed a suite of transcription and signaling factors that display polarized expression along the DV axis of the pharyngeal arches in skate. The overwhelming majority of genes discussed above share patterns of expression in the mandibular, hyoid and gill arches (fig. 9*A*). Together with previous reports of shared expression of core components of the pharyngeal arch DV patterning network in cartilaginous and bony fishes (Compagnucci et al. 2013; Gillis et al. 2013), and the fact that many genes involved in DV patterning of the jaw skeleton in zebrafish have comparable hyoid arch skeletal patterning functions, our findings point to a conserved transcriptional network patterning the DV axis of the mandibular, hyoid, and gill arches in the gnathostome crown group, and serial homology of the gnathostome jaw, hyoid, and gill arch skeleton. We additionally report distinct transcriptional features of the mandibular and gill arches in skate (fig. 9*B*), including dorsal mesenchymal expression of *six1*, e*ya1*, and *scamp5*, mandibular arch mesoderm-specific expression of s*ix2*, *tbx18*, and *pknox2*, hyoid/gill arch mesoderm-specific expression of *lhx9*, and the expression in developing gills of *foxl2*, *gcm2*, *wnt2b*, and *foxq1*. The aforementioned mesenchymal gene expression features could reflect mandibular arch-specific divergence from the ancestral pharyngeal DV patterning program, and could function downstream of global anteroposterior patterning mechanisms (e.g., the “Hox code” of the vertebrate head) and in parallel with local signals from oral epithelium to effect anatomical divergence of the mandibular arch skeleton (Hunt et al. 1991; Rijli et al. 1993; Couly et al. 1998, 2002; Hunter and Prince 2002), whereas mesodermal and endodermal gene expression features could underlie the evolution of arch-specific muscular and gill fates, respectively.

Cyclostomes (lampreys and hagfishes) are the most proximate living sister group to the gnathostomes, and the cyclostome pharyngeal endoskeleton and oral apparatus departs considerably from the condition seen in cartilaginous and bony fishes. Lampreys possess a muscular lower lip and lingual and velar cartilages that derived from the first pharyngeal (mandibular) arch, a muscular upper lip that derives largely from the premandibular domain, and a branchial “basket” consisting of a series of unjointed cartilaginous gill, epitrematic, and hypotrematic bars, derived from the hyoid and gill arches (Johnels 1948). Although this lamprey pharyngeal skeleton arises from embryonic tissue interactions and gene expression patterns that share some broad similarities with those giving rise to the pharyngeal endoskeleton of gnathostomes (reviewed by Square et al. 2017), notable embryological and molecular differences also contribute to the considerable pharyngeal anatomical disparity exhibited by cyclosomes and gnathostomes. For example, lampreys possess six *Dlx* genes of unclear orthology with those of gnathostomes (Myojin et al. 2001; Neidert et al. 2001; Kuraku et al. 2010)—and although these genes are expressed in a nested pattern in the mesenchyme of all pharyngeal arches (Cerny et al. 2010), this pattern differs from the broadly conserved “Dlx code” that has been described in various gnathostome taxa. Additionally, in the rostral pharynx of the lamprey, *Dlx*-expressing neural-crest-derived mesenchyme is not confined to the mandibular arch, but rather extends into the premandibular domain, and patterns of *Dlx* gene expression in this oral region differ from those seen in the posterior pharyngeal arches (Cerny et al. 2010; reviewed by Miyashita and Diogo 2016). Homology of the mandibular arch of cyclostomes and gnathostomes, as an embryological structure, is well established (Kimmel et al. 2001). However, despite classical and contemporary attempts to identity putative homologies between the mandibular arch-derived skeletons of cyclostomes and gnathostomes, it seems increasingly likely that such 1:1 correspondence between the jaw elements of gnathostomes and the oral skeleton of cyclostomes do not exist.

Rather, most developmental hypotheses of jaw evolution aim to explain the origin of the gnathostome jaw by modification of a cyclostome-like condition. Such scenarios include a heterotopic shift in epithelial-mesenchymal interactions restricting skeletogenic transcription factor expression to the mandibular arch (Shigetani et al. 2002), confinement of the embryonic progenitors of ancestrally distinct rostral pharyngeal skeletal elements to the mandibular arch, and subsequent assimilation of mandibular arch derivatives to segmented skeletal arrangement found in more caudal arches (Miyashita 2016), or co-option of a developmental mechanism promoting joint fate into a mandibular arch that is otherwise largely gnathostome-like in its DV patterning (Cerny et al. 2010). Importantly, these hypotheses are all predicated on the cyclostome-like pharyngeal skeleton reflecting an ancestral vertebrate condition. There are some paleontological data supporting this view, though these come in the form of inferred cyclostome-like skeletal conditions from casts of cranial nerve paths and muscle scars inside the dermal head shield of stem gnathostomes, and not from direct observation of endoskeletal preservation (Janvier 1996). Preservation of the cartilaginous skeletal elements of early vertebrates is rare, but has been reported for the Cambrian stem vertebrate *Metaspriggina walcotti* (Morris 2008), recently reconstructed as possessing seven paired gill bars, each segmented into bipartite dorsal and ventral elements (reminiscent of the epi- and ceratobranchials of crown gnathostomes) (Morris and Caron 2014). If this reconstruction reflects faithful preservation of the pharyngeal endoskeleton—and if the most rostral of these segmented bars is derived from the first pharyngeal arch—this would imply that a pharyngeal skeletal organization more closely resembling that of crown gnathostomes (i.e., with a serially repeated set of segmented skeletal derivatives arising from each pharyngeal arch) could, in fact, be plesiomorphic for vertebrates. It would follow that differences between cyclostome and gnathostome pharyngeal skeletons reflect cyclostome divergence from a plesiomorphic condition retained in gnathostomes (rather than vice versa), and that the pan-pharyngeal transcriptional program discussed above could have functioned to pattern the DV axis and to serially delineate pharyngeal skeletal segments not just in the last common ancestor of the gnathostome crown group, but more generally, in the last common ancestor of vertebrates.

## Materials and Methods

### Embryo Collection

*Leucoraja erinacea* embryos for mRNA ISH were collected at the Marine Biological Laboratory (Woods Hole, MA, USA). Embryos were fixed in 4% paraformaldehyde overnight at 4 °C, rinsed in phosphate-buffered saline (PBS), dehydrated stepwise into 100% methanol, and stored in methanol at −20 °C. Skate embryos were staged according to Ballard et al. (1993) and Maxwell et al. (2008).

### Gene Cloning and mRNA In Situ Hybridization Probe Synthesis

Cloned fragments of skate cDNAs were PCR amplified from total embryonic cDNA template using standard protocols. PCR products were isolated and purified using the MinElute Gel Extraction Kit (Qiagen) and ligated into the pGemT-easy Vector System (Promega). Resulting plasmids were transformed into JM109 *E. coli* (Promega) and prepared using a standard alkaline miniprep protocol. Insert sequences were verified by Sanger Sequencing (University of Cambridge, Department of Biochemistry). Linearized plasmid was used as a template for in vitro transcription of DIG-labeled riboprobes for mRNA ISH, using 10× DIG-labeled rNTP mix (Roche) and T7 RNA polymerase (Promega), according to manufacturers’ directions. Probe reactions were purified using the RNA Clean and Concentrator kit (Zymo Research).

### Histology and In Situ Hybridization

Paraffin embedding, sectioning, and ISHs on sections were performed as described previously (O'Neill et al. 2007; with modifications according to Gillis et al. 2012).

For wholemount in situ hybridizations (WMISH), embryos were rehydrated through a methanol gradient into diethylpyrocarbonate (DEPC)-treated PBS with 0.1% Tween-20 (100%, 75%, 50%, 25% methanol in DEPC-PBT), then treated with a 1:2,000 dilution of 10 mg/ml proteinase K in DEPC PBT for 15 min at room temperature. Following a rinse in DEPC-PBT, embryos were refixed in 4% PFA/DEPC-PBS for 15 min at room temperature and washed in DEPC-PBT again. Specimens were prehybridized in hybridization solution (5× SSC, 50% formamide, 1% SDS, 50 μg/ml yeast tRNA, 25 μg/ml heparin) for 1 h at room temperature. Hybridization was performed overnight at 70 °C with dig-labeled riboprobe diluted to 1 ng/μl in hybridization solution. Embryos were washed twice for 1 h each at 70 °C in wash solution 1 (50% formamide, 2×SSC, 1% SDS), twice for 30 min each at 70 °C in wash solution 3 (50% formamide, 1×SSC), then three times for 10 min at room temperature in MABT (0.1 M maleic acid, 150 mM NaCl, 0.1% Tween-20, pH 7.5). After blocking for 2 h at room temperature in 20% sheep serum + 1% Boehringer blocking reagent in MABT, embryos were incubated overnight at 4 °C with a 1:2,000 dilution of antidigoxigenin antibody (Roche) in blocking buffer. Embryos were then washed in MABT (two quick rinses then five 30-min washes), stored overnight in MABT at 4 °C and equilibrated in NTMT (100 mM NaCl, 100 mM Tris pH 9.5, 50 mM MgCl2, 0.1% Tween-20). The color reaction was initiated by adding BM Purple (Merck) to the embryos, and stopped by transferring to PBS. Embryos were rinsed once in PBS, postfixed in 4% PFA for 30 min, and graded into 75% glycerol in PBS for imaging.

For gelatin embedding, WMISH embryos were equilibrated in a 15% w/v gelatin solution in PBS at 50 °C for 1 h, before being poured into plastic molds, positioned for sectioning and left to cool. Gelatin blocks were then postfixed in 4% PFA at 4 °C for 4 days and rinsed in PBS. About 50 μm sections were cut using a Leica VTS1000 vibratome and mounted on Superfrost slides (VWR) using Fluoromount G (SouthernBiotech).

### RNAseq, De Novo Transcriptome Assembly, and Differential Gene Expression Analysis

Total RNA was extracted from upper mandibular arch (*n *= 10), lower mandibular arch (*n *= 6), upper gill arch 1 (*n *= 5), and lower gill arch 1 (*n *= 3) domains at stage (S)23/S24 and from upper mandibular arch (*n* = 6), lower mandibular arch (*n* = 6), upper gill arch 1 (*n *= 4), and lower gill arch 1 (*n* = 4) domains at S25/S26 (fig. 5*A*). Note that gill arch 1 refers to the third pharyngeal arch, and not the hyoid (second) arch. S23–S24 and S25–S26 span the expression of the *dlx* code, a key regulator of axial identity in the pharyngeal arches. In mouse, combinatorial *dlx* expression is observed in the mandibular and hyoid arch (Depew et al. 2002), whereas in zebrafish, *dlx* genes are expressed in a nested pattern in all pharyngeal arches (Talbot et al. 2010; Barske et al. 2020), though precise boundaries of combinatorial expression are somewhat difficult to identify in the caudal pharyngeal arches, owing to their relatively small size. Additionally, in zebrafish, it is not clear whether or how nested *dlx* gene expression patterns the epi- and ceratobranchial cartilages of the gill arches. Skates exhibit shared, nested expression *dlx* genes in the developing mandibular, hyoid, and gill arches, in a pattern that is largely reminiscent of the mouse mandibular arch, and it has been shown through lineage tracing that dlx gene expression boundaries correspond with anatomical boundaries in the differentiated skeleton (Gillis et al. 2013). Manual dissections of upper and lower arch primordia were therefore guided by morphological landmarks correlating with dorsal (*dlx1/2+*) and ventral (*dlx1-6+*) expression territories, as reported by Gillis et al. (2013).

Samples were preserved in RNAlater, total RNA was extracted using the RNAqueous-Micro Total RNA Isolation Kit (ThermoFisher), and library prep was performed using the Smart-seq2 (Picelli et al. 2014) with 10 cycles of cDNA amplification. S23/S24 and S25/S26 libraries were pooled and sequenced using the HiSeq4000 platform (paired-end sequencing, 150-bp read length) at the CRUK genomics core facility (University of Cambridge, Cancer Research UK Cambridge Institute). In addition to the above, libraries from the dorsal mandibular arch (*n* = 5), ventral mandibular arch (*n* = 5), dorsal gill arch (*n *= 5), and ventral gill arch (*n* = 5) domains of S29 skate embryos were prepared as described above, and sequenced using the NovaSeq 6000 (paired-end sequencing, 150-bp read length) at Novogene Co., Ltd. Reads from these libraries were included in our de novo transcriptome assembly, but are not analyzed further in the current work.

A total of 2,058,512,932 paired raw reads were used. Low-quality read and adapter trimming were conducted with Trim Galore! (0.4.4) with the quality parameter set to 30 and phred cut-off set to 33. Reads shorter than 65 bp were discarded. After trimming adapters and removing low-quality reads a total of 1,348,098,076 reads were retained. Normalization (max coverage 30) reduced this to a further 54,346,196 reads. The de novo assembly based on these reads was generated using Trinity 2.6.6 with default parameters (Grabherr et al. 2011; Haas et al. 2013). The N50 is 1,009 bp, and the Ex90N50 (the N50 statistic computed as usual but considering only the topmost highly expressed transcripts that represent 90% of the total normalized expression data, meaning the most lowly expressed transcripts are excluded) is 1906 bp (supplementary table S1, Supplementary Material online). Postassembly quality control was carried out using Trinity’s toolkit or gVolante (supplementary fig. S3 and table S1, Supplementary Material online).

Trinity transcript quantification was performed alignment-free using salmon (Patro et al. 2017) to estimate transcript abundance in TPM (transcripts per kilobase million). The genes differentially expressed along the DV axis within each arch, or across the anterior–posterior axis between dorsal and ventral elements of each arch, were screened for using edgeR with a cut-off of FDR (false discovery rate) ≤0.05 (supplementary table S2, Supplementary Material online for gene numbers, supplementary table S3, Supplementary Material online for candidates for validation, supplementary tables S4–S7, Supplementary Material online for stages 24/25, and supplementary tables S8–S11, Supplementary Material online for stages 25/26). edgeR was used to perform a negative binomial additive general linear model with a quasi-likelihood *F*-test, and model design accounted for repeated sampling of tissues from the same individual and *P* values were adjusted for multiple testing using the Benjamin–Hochberd method to control the FDR (FDR ≤0.05) (supplementary fig. S3*A*–*D*, Supplementary Material online) (Johnson et al. 2007). The screened transcripts were putatively annotated based on sequence similarity searches using blastx against Uniprot (http://www.uniprot.org/).

## Supplementary Material

Supplementary data are available at *Molecular Biology and Evolution* online.

## Supplementary Material

msab123_Supplementary_Data
