# Supplementary material for: Conserved and unique transcriptional features of pharyngeal arches in the skate (Leucoraja erinacea) and evolution of the jaw
Source: Mol Biol Evol. 2021 Apr 27;38(10):4187–204. doi: 10.1093/molbev/msab123 (PMC8476176; doi:10.1093/molbev/msab123)
Supplement: msab123_Supplementary_Data [file msab123_supplementary_data.zip › Table_S1.docx]

| **Reads** |  |  |
| --- | --- | --- |
|  | Raw reads | 2058512932 |
|  | Clean reads | 1348098076 |
|  | Normalised reads | 54346196 |
| **Assembly** |  |  |
|  | Total Trinity transcripts | 549531 |
|  | Total Trinity genes | 364865 |
|  | % GC | 46.26 |
| **Statistics based on all isoforms per gene** |  |  |
|  | N50 | 1009 |
|  | Ex90N50 | 1906 |
|  | Median length | 635 |
|  | Average length | 818 |
| **Read representation** | Average read content per library | 89% |
| **BUSCO v3, Core Vertebrate Gene set** | Total # of core genes queried: | 233 |
|  | # of core genes detected |  |
|  | Complete: | 205 (87.98%) |
|  | Complete + partial: | 225 (96.57%) |
|  | # of missing core genes: | 8 (3.43%) |
