# Supplementary material for: Conserved and unique transcriptional features of pharyngeal arches in the skate (Leucoraja erinacea) and evolution of the jaw
Source: Mol Biol Evol. 2021 Apr 27;38(10):4187–204. doi: 10.1093/molbev/msab123 (PMC8476176; doi:10.1093/molbev/msab123)
Supplement: msab123_Supplementary_Data [file msab123_supplementary_data.zip › Table_S2.docx]

|  | Gill arch S23_24 | Mandibular arch S23_24 | Gill arch S25_26 | Mandibular arch S25_26 |
| --- | --- | --- | --- | --- |
| Dorsal | 10 | 57 | 11 | 46 |
| Ventral | 9 | 54 | 21 | 44 |
|  |  |  |  |  |
|  | Dorsal S23_24 | Ventral S23_24 | Dorsal S25_26 | Ventral S25_26 |
| Gill arch | 238 | 83 | 45 | 919 |
| Mandibular arch | 200 | 105 | 71 | 691 |
